# Antitumor- and apoptosis-inducing effects of pomolic acid against SK-MEL-2 human malignant melanoma cells are mediated via inhibition of cell migration and sub-G1 cell cycle arrest

**DOI:** 10.3892/mmr.2021.12120

**Published:** 2021-04-26

**Authors:** Tian-Hang Li, Hong-Xia Yan

Mol Med Rep 17: 1035-1040, 2018; DOI: 10.3892/mmr.2017.7977

Following the publication of the above paper, a concerned reader drew to the Editor's attention that several figures (Figs. 3–8 inclusive) contained apparent anomalies, including repeated patternings of data within the same figure panels.

After having conducted an independent investigation in the Editorial Office, the Editor of *Molecular Medicine Reports* has determined that the above paper should be retracted from the Journal on account of a lack of confidence concerning the originality and the authenticity of the data. The authors were asked for an explanation to account for these concerns, but the Editorial Office never received any reply. The Editor regrets any inconvenience that has been caused to the readership of the Journal.

